# PAP Versus DIEP Flap Breast Reconstruction: Current Evidence and the Unresolved Question of Timing and Oncologic Safety—A Narrative Review

**DOI:** 10.3390/medsci14020295

**Published:** 2026-06-06

**Authors:** Maximilian Vlad Muntean, Radu Alexandru Ilieș, Vlad Alexandru Gâta, Ștefan Țîțu, Ioan Constantin Pop, Alex Victor Orădan, Gerald Gheorghe Filip, Roxana Pintican, Nicoleta Zenovia Antone, Patriciu Andrei Achimaș-Cadariu

**Affiliations:** 1 Department of Plastic and Reconstructive Surgery, “Iuliu Hațieganu” University of Medicine and Pharmacy, 400012 Cluj-Napoca, Romania; 2 Department of Plastic Surgery, “Prof. Dr. Ion Chiricuță” Institute of Oncology, 400015 Cluj-Napoca, Romania; 3 Faculty of Medicine, “Iuliu Hațieganu” University of Medicine and Pharmacy, 400012 Cluj-Napoca, Romania; 4 Department of Oncological Surgery and Gynecological Oncology, “Iuliu Hațieganu” University of Medicine and Pharmacy, 400012 Cluj-Napoca, Romania; 5 Department of Surgical Oncology, “Prof. Dr. Ion Chiricuță” Institute of Oncology, 400015 Cluj-Napoca, Romania; 6 Department of Surgery, Ponderas Academic Hospital, 014142 Bucharest, Romania; 7 Department of Radiology, “Iuliu Hațieganu” University of Medicine and Pharmacy, 400012 Cluj-Napoca, Romania; 8 Department of Oncology, “Prof. Dr. Ion Chiricuță” Institute of Oncology, 400015 Cluj-Napoca, Romania

**Keywords:** breast reconstruction, DIEP flap, PAP flap, oncologic outcomes, reconstructive timing

## Abstract

**Background/Objectives**: Deep inferior epigastric perforator (DIEP) flap reconstruction represents the gold standard for autologous breast reconstruction, while profunda artery perforator (PAP) flap reconstruction has developed as a reliable alternative, particularly in patients with low body mass index or inadequate abdominal tissue. Even though several comparative studies have evaluated surgical and patient-reported outcomes between PAP and DIEP flaps, evidence regarding reconstructive timing, oncologic safety, and interactions with adjuvant therapies remains scarce, especially for PAP reconstruction. **Methods**: A narrative review of the literature was conducted using PubMed. Studies assessing PAP and DIEP flap breast reconstruction were included, with particular focus on surgical outcomes, patient-reported outcomes, reconstructive timing (immediate or delayed reconstruction), oncologic safety, recurrence, and the effects of radiotherapy and chemotherapy. Comparative studies, cohort studies, systematic reviews, and meta-analyses were synthesized through a narrative review. **Results**: Twenty studies were included. Comparative evidence showed similar flap survival rates and overall patient satisfaction between the two methods, with flap success rates approaching 98–100%. PAP reconstruction was associated with increased donor-site wound complications and, in some studies, increased fat necrosis rates, while long-term patient-reported and aesthetic outcomes remained equivalent between techniques. In contrast to the relatively limited PAP literature, DIEP reconstruction has been widely studied in terms of reconstructive timing and oncologic safety. Current evidence indicates that immediate DIEP reconstruction does not increase the risk of flap loss, major complications, or recurrence in comparison with delayed reconstruction and might optimize early postoperative recovery and patient-reported outcomes. Nevertheless, none of the identified studies directly compared PAP and DIEP reconstruction with respect to immediate versus delayed timing, exposure to radiotherapy or chemotherapy, or long-term oncologic outcomes. **Conclusions**: PAP flap appears to represent a reliable alternative to DIEP flap reconstruction. However, major gaps in the literature persist involving PAP reconstruction in oncologic and timing-related settings. Future prospective multicenter studies that directly compare PAP and DIEP flaps according to reconstructive timing, exposure to adjuvant therapy, recurrence, and patient-reported outcomes are warranted to establish evidence-based reconstructive strategies for oncologic breast reconstruction.

## 1. Introduction

Autologous breast reconstruction using free flaps has become a key component of postmastectomy breast cancer care because it offers valuable benefits regarding quality of life, body image, and patient satisfaction following oncologic treatment [1,2,3]. Even though there are multiple available autologous techniques, the deep inferior epigastric perforator (DIEP) flap is broadly considered the current gold standard for multiple reasons: its reliable vascular anatomy, high flap survival rates, and favorable patient-reported outcomes [1,2,4]. However, not every patient is a suitable candidate for abdominal-based reconstruction: individuals with a low body mass index, those with previous abdominal surgery, patients with inadequate abdominal tissue volume, or patients for whom minimizing abdominal donor-site morbidity is an important consideration. In this context, another reconstructive option using the profunda artery perforator (PAP) flap has recently emerged as an alternative with an expanding clinical adoption [5,6,7,8].

Several studies have highlighted that PAP flap reconstruction can lead to equivalent flap survival and patient-reported outcomes compared to DIEP reconstruction, justifying its role as an efficient microsurgical option in appropriately selected cases [1,2,3,9]. Considering that the PAP flap has been recommended to patients who are not ideal candidates for abdominal donor sites, (including thin patients and those with previous abdominal surgical interventions) [4,5,6], the existing literature on this topic focuses on the comparison between PAP and DIEP flap, in terms of reconstructive results, donor-site morbidity, aesthetic results, and patient satisfaction [1,2,3,7].

Even though there is a growing body of evidence in favor of the reconstruction using the PAP flap, comparative studies remain primarily centered on surgical and patient-reported outcomes [1,2,4,5,6,8,10]. Until now, no identified study has directly compared PAP and DIEP flaps with the aim of discussing reconstructive timing, specifically immediate versus delayed reconstruction. Furthermore, they do not assess oncologic outcomes like local recurrence, disease progression, or the influence of reconstruction timing on adjuvant therapies. This limitation represents a gap in the existing literature of reconstructive surgery, mostly because therapeutic strategies for breast cancer continue to evolve toward increasingly individualized and multidisciplinary approaches.

On the other hand, the literature on DIEP flap reconstruction has broadened beyond reconstructive outcomes. The differences between immediate and delayed DIEP reconstruction with respect to complications, patient-reported outcomes, and perioperative management were investigated by multiple studies [11,12,13,14,15,16,17]. Moreover, there is evidence on the oncologic safety of DIEP reconstruction (including recurrence patterns) and even the potential influence of reconstruction timing depending on cancer-related outcomes [14,15,16,17,18,19]. Nevertheless, some studies have questioned the effects and impact of radiotherapy and chemotherapy on DIEP flap outcomes, underscoring the importance of integrating reconstructive timing with oncologic treatment planning [14,15,16].

Consequently, comparable evidence addressing all these aforementioned aspects in the field of PAP flap reconstruction remains virtually absent. The lack of studies comparing immediate and delayed PAP flap breast reconstruction, including their surgical characteristics, oncologic outcomes, and interactions with adjuvant therapies, significantly limits the development of evidence-based reconstructive algorithms for patients undergoing PAP flap reconstruction. PAP reconstruction continues to rise in popularity and gain clinical acceptance. Therefore, understanding how reconstructive timing and oncologic therapies influence surgical and oncologic outcomes is essential for appropriate patient selection, perioperative decision-making, and long-term management. The aim of the current review is to synthesize the available evidence regarding breast reconstruction using PAP and DIEP flap, with particular focus on comparative reconstructive outcomes, reconstructive timing, oncologic safety, and treatment-related outcomes. By integrating the existing evidence on DIEP reconstruction with the currently limited PAP-related data, this review also aims to identify the current knowledge gaps and formulate clinically relevant hypotheses and future research directions regarding immediate versus delayed PAP flap reconstruction.

## 2. Literature Search Strategy and Study Selection

This study is a narrative review with a structured literature search. The search was conducted using PubMed to find the studies evaluating autologous breast reconstruction with PAP, respectively, with DIEP flaps, with additional focus on reconstruction timing and oncologic outcomes. The primary search strategy combined terms that were related to PAP flaps, DIEP flaps, and breast reconstruction (70 results):

(“PAP flap” OR “profunda artery perforator” OR “profunda femoris artery perforator”) AND (“DIEP flap” OR “deep inferior epigastric perforator”) AND (“breast reconstruction”).

Additionally, another more targeted search was performed to assess reconstructive timing and oncologic or treatment-related outcomes (68 results):

(“DIEP flap” AND (“immediate” OR “delayed”) AND (“breast reconstruction”)) AND (“radiotherapy” OR “chemotherapy” OR “recurrence” OR (“oncologic outcomes”)).

The reference lists of the included studies and key reviews discussing PAP and DIEP reconstruction, together with alternative donor sites, were then screened manually to identify additional relevant publications. Through this citation-based approach, additional studies not indexed in PubMed were also identified and considered for inclusion.

Afterwards, selection criteria for the narrative synthesis were established and are presented in the following paragraphs.

Studies were considered eligible if they enrolled adult women who underwent autologous breast reconstruction following breast oncologic surgery or prophylactic mastectomy. Studies evaluating DIEP, PAP, or alternative perforator flaps like lumbar artery perforator (LAP) flaps that were used as comparator techniques were included.

The following outcome categories were taken into consideration:

- Surgical outcomes: fat necrosis, flap loss, donor-site and recipient-site complications, and reinterventions.
- Patient-reported outcomes (PROs): questionnaires and other validated patient-reported outcome evaluation instruments.
- Oncologic and timing-related outcomes: recurrence, effects of immediate versus delayed reconstruction, and radiotherapy- or chemotherapy-related outcomes.

Eligible study designs comprised randomized controlled trials, prospective and retrospective cohort studies, case–control studies, systematic reviews, and meta-analyses.

Single case reports, studies that assessed non-breast reconstructive indications, technical notes without outcome reporting, and expert-opinion articles were excluded unless they specifically contributed relevant information regarding PAP flap utilization or donor-site selection.

From each eligible study, data concerning study design, sample size, type of flaps, reconstructive timing (immediate or delayed, if available), the use of adjuvant treatments such as radiotherapy or chemotherapy, surgical complications, outcomes reported by patients, and oncologic endpoints were extracted.

Because of the heterogeneity of study designs and the limited existence of PAP-specific data on reconstruction timing and oncologic outcomes, a narrative review was performed.

For DIEP reconstruction, evidence from large cohort studies and meta-analyses exploring timing-related and oncologic outcomes was integrated in order to contextualize current evidence and identify knowledge gaps relevant to reconstruction using the PAP flap.

**Figure 1:**
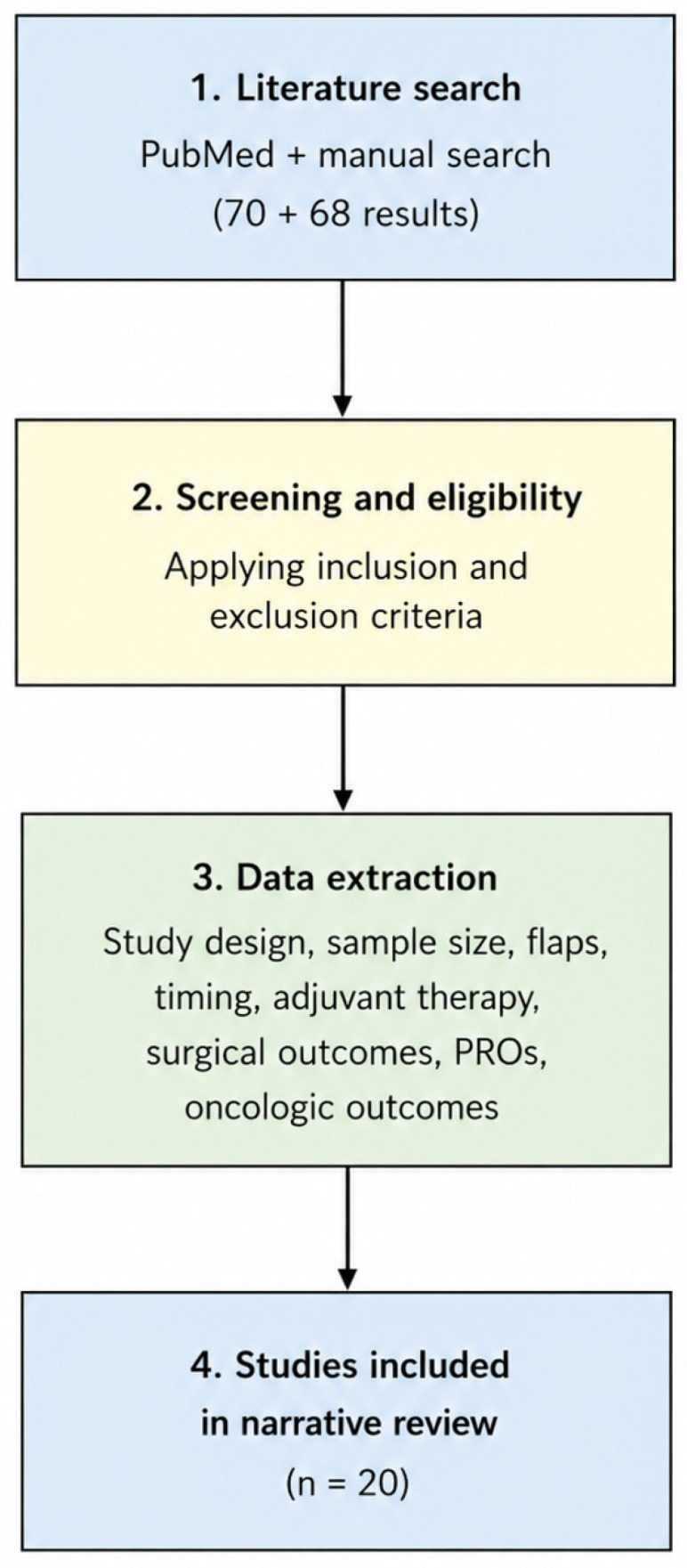
Flowchart of the literature search, study selection, and narrative synthesis process. PROs = patient-reported outcomes.

In total, 20 studies were identified and further included in the analysis (Figure 1), encompassing comparative PAP versus DIEP studies, DIEP timing analyses, and oncologic or treatment-related outcome investigations.

## 3. Results

### 3.1. Overview of Included Studies

The studies included in the narrative review, according to their primary research focus, are presented in Table 1. Some studies were assigned to multiple categories, depending on their topic and focus.

In most studies, comparisons were made between PAP and DIEP flaps or other alternative donor-site strategies for autologous breast reconstruction, representing the largest body of evidence identified in the reviewed literature (11 studies). These papers primarily discussed surgical complications, survival of flaps, donor-site morbidity, aesthetic outcomes, and patient-reported outcomes linked to reconstruction with PAP and DIEP, respectively [1,2,3,4,5,6,7,8,9,10,20].

The impact of reconstructive timing in breast reconstruction with DIEP flap was the focus area of the second group of studies, specifically comparing immediate and delayed reconstructions (8 studies). These studies explored differences regarding complications, perioperative outcomes, and patient-reported measures between the two timing strategies [11,12,13,14,15,16,17,18].

The third group included several studies that investigated oncologic outcomes and treatment-related outcomes linked to autologous breast reconstruction (7 studies). These papers reported the recurrence, oncologic safety, and the impact of radiotherapy and chemotherapy on reconstructive outcomes, together with timing considerations [11,13,14,15,16,18,19].

These findings will be presented in the following sections in detail, organized according to outcome categories.

On the whole, the table shows that even if some evidence regarding DIEP timing and oncologic outcomes exists, PAP-related evidence in these contexts is more limited. The current literature, which is available on this subject, predominantly concentrates on reconstructive and patient-reported outcomes, underscoring the lack of studies evaluating immediate versus delayed reconstruction with PAP flap and long-term oncologic implications.

### 3.2. Direct Comparisons Between PAP and DIEP Flaps

Six comparative studies evaluated surgical outcomes, complications, patient-reported outcomes, and aesthetic results between PAP and DIEP flap breast reconstruction [1,2,4,6,8,10], as represented in Table 2.

The largest retrospective comparative study was based on 677 patients who underwent autologous breast reconstruction, split into two groups: 559 DIEP flaps and 118 PAP flaps [1]. Patients who underwent PAP reconstruction were generally younger and had smaller breast volumes (*p* < 0.001). No statistically significant differences were found in terms of operative time, early or late reinterventions, or overall surgical complications, justifying the role of PAP reconstruction as a valid primary option for reconstruction, rather than a secondary alternative [1].

In a prospective non-blinded two-armed cohort study including 157 patients, a total of 207 autologous breast reconstruction procedures were reported, with 129 patients (82.1%) undergoing DIEP flap reconstruction and 21 patients (13.3%) undergoing PAP flap reconstruction. Bilateral reconstruction was performed more frequently in the DIEP group (37 patients, 28.6%), while only one PAP case (5%) was bilateral. The remaining PAP cases involved stacked flap techniques for unilateral reconstruction [2]. PAP flaps showed significantly shorter ischemia times when compared with DIEP flaps (55.29 ± 15.59 min vs. 69.52 ± 21.74 min, *p* = 0.014); conversely, donor-site wound dehiscence occurred more commonly in the PAP group (*p* = 0.014), while no other major differences regarding perioperative outcomes were reported [2].

A prospective matched cohort study included 36 patients and compared long-term outcomes after DIEP and PAP flap reconstruction [4]. BREAST-Q analysis showed no statistically significant differences between groups in the physical well-being of the breast, physical well-being of the donor site, or satisfaction with breasts domains, corresponding to the respective questionnaire sections. Donor-site scar assessment was additionally performed using the Patient and Observer Scar Assessment Scale (POSAS), a validated scar evaluation instrument, demonstrating comparable scar quality between abdominal and thigh donor sites. Aesthetic evaluation of reconstructed breasts by independent surgeons also revealed no significant differences between techniques. In general, DIEP and PAP flaps showed similar long-term patient-reported and aesthetic outcomes [4].

A propensity-matched trilateral comparison involving DIEP, PAP, and LAP flaps (50 cases in each group) demonstrated comparable long-term patient satisfaction across different groups, even though complication patterns differed between donor sites [6]. PAP reconstruction was linked to higher rates of donor-site wound complications and infections, whereas DIEP reconstruction had higher rates of breast wound complications and flap necrosis [6].

Another retrospective single-center comparative study of 85 PAP and 122 DIEP flaps reported shorter operative times, with faster ambulation postoperatively in the PAP flap reconstruction group. Follow-up duration was equivalent between groups, with 11.6 ± 5.8 months in the PAP cohort and 11.1 ± 5.8 months in the DIEP cohort, without a statistically significant difference (*p* = 0.621). The DIEP flap group had a higher mean body mass index compared to PAP recipients. BREAST-Q scores were higher in the DIEP group [8]. Despite these differences, the authors highlighted the substantial reconstructive potential of PAP flaps [8].

A retrospective ultrasound-based study evaluating fat necrosis included 31 PAP and 99 DIEP breast reconstructions [10]. No total flap losses occurred in either group. However, PAP reconstruction demonstrated higher donor-site morbidity and increased rates of fat necrosis when compared with DIEP reconstruction [10].

Direct comparative evidence demonstrated similar flap survival and global patient satisfaction between PAP and DIEP reconstruction [1,2,4,6,8,10].

In particular, PAP reconstruction was shown to be associated with increased rates of donor-site complications and fat necrosis, while DIEP reconstruction generally demonstrated slightly superior or even comparable patient-reported and aesthetic outcomes.

### 3.3. Outcomes Focused on PAP Reconstruction

Four studies were included that evaluated PAP flap reconstruction outcomes; however, not all were purely single-arm analyses, as some also included comparative data with DIEP and/or LAP flaps [3,7,9,20], as summarized in Table 3.

A meta-analysis based on 24 studies and a total of 1612 PAP flap reconstructions demonstrated an overall success rate of the flap equal to 99.6%, with low rates of total flap loss and recipient-site complications [3]. Donor-site wound dehiscence was the most frequently reported complication. Comparative analyses with DIEP reconstruction, when available, revealed no significant differences in terms of flap failure, fat necrosis, or rates of donor-site dehiscence [3].

Moreover, a systematic review comparing PAP, stacked PAP, LAP, and DIEP reconstructions analyzed more than 23,000 DIEP flaps and a total of 745 PAP reconstructions [7]. PAP flap success rates were equivalent to DIEP reconstruction, although stacked PAP and LAP techniques showed lower flap survival in comparison to single PAP or DIEP procedures [7].

Prospective single-center data derived from the assessment of 116 PAP flap reconstructions in 86 patients (64 were unilateral and 22 bilateral) showed low donor-site complication rates: hematoma (1.7%), seroma (2.6%), fat necrosis (1.7%), and wound dehiscence (2.6%), with no complications such as flap thrombosis or flap loss [9]. Significant postoperative improvements regarding BREAST-Q scores, particularly satisfaction with breast appearance, were observed at 12 months following breast reconstruction [9].

A narrative clinical practice review summarized the current evidence regarding PAP flap anatomy, surgical techniques, technical refinements, and complication profiles, supporting the fact that PAP reconstruction represents a strong alternative in patients who are not suitable for abdominal-based reconstruction [20].

These findings confirmed that PAP reconstruction can achieve high flap survival rates, together with favorable patient-reported results, supporting its role as a useful alternative autologous reconstructive option in carefully selected cases [3,7,9,20].

### 3.4. Broader Comparative Reviews of Autologous Reconstruction

One narrative review performed a broader comparison between DIEP, PAP, and latissimus dorsi flap reconstruction [5]. DIEP reconstruction was viewed as the gold standard for autologous breast reconstruction, due to its favorable balance between reconstructive outcomes and donor-site morbidity. On the other hand, reconstruction with the PAP flap was regarded as an increasingly important option for patients lacking sufficient abdominal tissue, whereas latissimus dorsi reconstruction remained a versatile and reliable option that was commonly combined with other methods, such as the use of implants or sessions of fat grafting [5].

### 3.5. DIEP Timing, Oncologic Safety, and Long-Term Outcomes

Nine studies analyzed characteristics like reconstructive timing, oncologic safety, recurrence, and treatment-related outcomes in breast reconstruction with DIEP flap [11,12,13,14,15,16,17,18,19], as shown in Table 4.

#### 3.5.1. Immediate Versus Delayed DIEP Reconstruction

The largest multicenter European study was based on 3926 patients undergoing 4577 DIEP flap reconstructions and showed similar rates of total and partial flap loss when comparing immediate and delayed reconstruction [11]. Immediate reconstruction was linked to fewer revision procedures, a shorter hospital stay, and earlier mobilization postoperatively [11].

A retrospective risk-factor analysis of 802 DIEP flap cases identified two independent predictors of postoperative complications: the prolonged operative duration and the immediate reconstruction [12].

Conversely, a prospective Vietnamese cohort evaluated immediate DIEP reconstruction and showed a high rate of flap success, together with favorable patient satisfaction rates and acceptable timing in relation to adjuvant chemotherapy initiation [13].

A meta-analysis analyzing a total of 5784 DIEP reconstructions reported significantly fewer complications of wound healing following immediate reconstruction in comparison with delayed reconstruction, whereas the rates of hematoma, infection, fat necrosis, and flap loss were similar between the compared groups [18].

The multicenter UMBRELLA cohort study included longitudinal patient-reported outcome data and demonstrated improved early body image and physical functioning in patients undergoing immediate reconstruction compared with those undergoing delayed reconstruction, although long-term BREAST-Q and European Organization for Research and Treatment of Cancer quality-of-life questionnaire (EORTC) outcomes turned equivalent over time [15]. In contrast, patients who underwent immediate reconstruction reported higher rates of fibrosis and movement restriction [15].

#### 3.5.2. Oncologic Outcomes and Recurrence

A cohort study including 862 patients and a total of 919 DIEP reconstructions assessed the oncologic recurrence after immediate and delayed reconstruction [16]. Local recurrence rates remained decreased in both groups, although adjusted analyses highlighted a potentially increased risk of recurrence in the delayed reconstruction group. The authors acknowledged the likelihood of selection bias and advised cautious interpretation [16].

Another study assessing local recurrence after DIEP flap breast reconstruction found a low overall recurrence prevalence and showed that most local recurrences might be surgically treated with preservation of the reconstructed DIEP flap [19]. The authors concluded that DIEP reconstruction did not tend to increase the risk of recurrence in comparison with mastectomy alone [19].

#### 3.5.3. Radiotherapy, Chemotherapy, and Reconstructive Outcomes

The influence of radiotherapy and chemotherapy on DIEP reconstruction was discussed in several studies [13,14,15,17,18].

Increased donor-site and recipient-site complications in patients receiving multimodal oncologic treatment were demonstrated in a single-center analysis investigating combined radiotherapy and chemotherapy [14]. Ischemia time was independently linked to recipient-site complications [14].

An extensive longitudinal comparison between tissue expander/implant-based breast reconstruction and DIEP reconstruction demonstrated significantly lower long-term major rates of complication in DIEP patients, particularly in patients who received radiotherapy [17]. No major differences were recorded in chemotherapy-only subgroups [17].

## 4. Discussion

### 4.1. Summary of the Main Evidence

In the included studies, the multiple comparisons of the two reconstruction strategies (PAP versus DIEP) showed equivalent outcomes concerning flap survival and long-term patient satisfaction, supporting the fact that PAP reconstruction appears to be a reliable alternative to DIEP reconstruction [1,2,4,6,8,10]. PAP reconstruction was more consistently performed in younger and lower-BMI patients, but in several studies, it was associated with donor-site morbidity and fat necrosis.

Meta-analyses and systematic reviews that were focused on PAP reconstruction confirmed flap success rates not far from those reported for DIEP reconstruction, with low rates of major complications, overall [3,7]. However, nearly all the available evidence on PAP reconstruction concentrated on reconstructive and patient-reported outcomes.

On the other hand, DIEP reconstruction has been extensively studied in terms of the timing of reconstruction, oncologic safety, recurrence, and even the influence of radiotherapy and chemotherapy [11,12,13,14,15,16,17,18,19].

Immediate DIEP reconstruction generally showed acceptable complication profiles, low recurrence rates, and a favorable integration with adjuvant oncologic therapy.

Altogether, the available evidence from the studied literature confirms that, generally, PAP reconstruction is a safe and effective alternative to DIEP reconstruction, while simultaneously underscoring the absence of PAP-specific evidence in terms of immediate versus delayed reconstruction, oncologic safety, recurrence, and potential interactions with adjuvant therapies. This represents the principal remaining evidence gap identified in the current literature.

### 4.2. PAP Flap as an Established Alternative to DIEP Reconstruction

Current evidence indicates that PAP flap breast reconstruction has emerged as a secondary reconstructive option and has become a reliable alternative to DIEP reconstruction in appropriately selected cases. Comparative studies demonstrated equivalent flap survival and comparable rates of perioperative complications and surgical morbidity between PAP and DIEP flaps, with reported success rates approaching 98–100% across retrospective cohort studies, prospective studies, and even reviews (narrative and systematic) and meta-analyses [1,2,3,4,5,6,7,8,9,10].

Even though the global reconstructive success appears comparable, the patterns regarding donor-site morbidity are different between the two techniques because of the distinct anatomical donor regions that are involved. DIEP reconstruction is related to abdominal donor-site morbidity, whereas PAP reconstruction could present a higher incidence of complications involving the thigh wound and dehiscence of the donor site reported in some cohorts [2,3,6,8,10]. Elevated rates of fat necrosis following breast reconstruction with PAP flaps were also reported in a study [10]. However, these differences did not appear to significantly have an impact on long-term reconstructive satisfaction.

The aesthetic outcomes, together with other long-term patient-reported outcomes, were, on the whole, equivalent between the two reconstructive methods [2,4,6,9]. BREAST-Q analyses showed similar scores in the following questionnaire domains: satisfaction with breast appearance, general well-being of the donor sites, and overall quality of life, reinforcing the concept that the selection of flaps might be dependent on the anatomy of the patient, lifestyle characteristics, and reconstructive preferences rather than major discrepancies in reconstructive efficacy [4,5]. Therefore, PAP reconstruction currently represents an option for reconstruction, particularly in the category of patients with decreased BMI or insufficient availability of abdominal tissue [1,3,5,7,20].

In this context, technical modifications of the DIEP flap, like the lipo-DIEP approach, have been developed to expand the reconstructive indications in patients having limited abdominal donor-site volume [21]. By combining standard DIEP flap harvest with immediate autologous fat grafting, this technique may enhance achievable breast volume while maintaining the benefits of autologous reconstruction [21]. However, although early evidence indicates favorable safety and volumetric outcomes, its broader clinical implications, particularly in terms of standardization and long-term oncologic and reconstructive outcomes, remain to be further defined.

### 4.3. Established Evidence Regarding DIEP Timing and Oncologic Safety

In contrast to the comparatively scarce PAP literature, DIEP flap reconstruction has been extensively studied with respect to reconstructive timing, oncologic safety, and the impact of adjuvant therapies. Multiple large cohort studies and meta-analyses provided evidence that immediate DIEP reconstruction does not increase the rates of flap loss or the risk of other major complications when compared with delayed reconstruction [11,13,18]. Even more, immediate reconstruction could offer several advantages, like reduced wound-healing complications, shorter hospitalization, and generally an improved early recovery postoperatively [11,18].

Interestingly, the relation between immediate reconstruction and radiotherapy remains complex. While long-term patient-reported outcomes following immediate and delayed DIEP reconstruction tend to be similar, immediate reconstruction has been associated with improved early body image and better short-term physical functioning; however, when followed by post-mastectomy radiotherapy (PMRT), it may also be associated with increased fibrosis and subsequent movement restriction [15]. In addition, it was demonstrated that radiotherapy and chemotherapy have an impact on reconstructive outcomes, mostly in multimodal treatment settings [14]. Longer times of ischemia and combined exposure to radiotherapy and chemotherapy were found to be relevant risk factors for recipient-site complications [12,14].

The safety of DIEP reconstruction is supported by the available oncologic evidence. Reported local recurrence rates remain decreased following the reconstruction, and the consulted studies do not imply an increased oncologic risk that is attributable to the reconstructive procedure itself [16,19]. All these findings provide a relatively solid evidence base guiding the timing strategies and perioperative planning in the case of DIEP reconstruction.

### 4.4. The Major Knowledge Gap: PAP Flap Reconstruction in Relation to Oncologic Treatment and Reconstructive Timing (Immediate vs. Delayed)

Although PAP flap reconstruction is increasingly accepted and supported by substantial evidence regarding its reconstructive reliability, significant gaps remain regarding its role within oncologic breast reconstruction pathways. While comparative data assessing surgical outcomes and patient-reported measures between PAP and DIEP flaps are available [1,2,3,4,5,6,7,8,9,10], there was no evidence to address reconstructive timing, patterns of recurrence, and the complex interactions between PAP reconstruction and adjuvant oncologic therapies.

None of the included studies compared PAP and DIEP methods of reconstruction within the same oncologic population, via stratifying patients into immediate versus delayed reconstruction, exposure to PMRT, administration of chemotherapy, or oncologic stage. Likewise, no available study assessed recurrence rates, oncologic survival, or the interval from mastectomy to initiation of adjuvant therapy in relation to flap type and reconstructive timing.

Consequently, the current decision-making process of reconstructing with PAP flaps is largely based on extrapolation derived from DIEP-based evidence. However, it remains unclear whether PAP reconstruction shares the same profile of oncologic safety and radiotherapy-related behavior as DIEP reconstruction. Considering the anatomical and donor-site differences between abdominal and thigh-based flaps, distinct profiles of complications or therapy-induced interactions could exist, particularly in cases with PMRT or multimodal oncologic treatment.

### 4.5. Limitations of the Current Review

A limitation of this review is that the literature search was restricted to PubMed only. Consequently, relevant studies indexed in other databases such as Embase, Scopus, Web of Science, and the Cochrane Library may not have been captured. This may introduce a risk of selection bias and limit the comprehensiveness of the evidence synthesis.

An additional limitation of this review is the heterogeneity of the included studies in terms of design, patient populations, and reported outcome measures, which may reduce the comparability of results across studies.

Furthermore, the majority of included evidence originates from retrospective cohorts, which are inherently subject to selection and reporting bias.

Finally, variability in the definition and reporting of key outcomes, particularly those related to reconstructive timing and oncologic endpoints, limited the ability to perform a quantitative synthesis and necessitated a narrative approach.

### 4.6. Clinical Implications and Future Research Directions

Current evidence supports multiple clinically relevant hypotheses that could be further explored in future studies.

PAP and DIEP reconstruction could demonstrate comparable oncologic safety and equivalent timelines in terms of the initiation of adjuvant therapies when they are used in immediate breast reconstruction settings. Furthermore, the different characteristics and qualities of the donor site in the case of PAP flaps might have a different impact on complication patterns in patients who received radiotherapy, potentially influencing the strategies of reconstructive timing. In the end, PAP reconstruction can represent a particularly valuable option for low-BMI patients who require PMRT, possibly allowing immediate reconstruction while maintaining favorable patient-reported outcomes.

Future research should concentrate on prospective multicenter studies that directly compare PAP and DIEP reconstruction in oncologic populations. Stratification in accordance with reconstructive timing, PMRT exposure, chemotherapy, tumor stage, and patient BMI would be mandatory to clarify the existing interaction between flap selection and oncologic therapeutic pathways. Additionally, propensity-matched analyses using existing institutional databases could provide valuable preliminary evidence on the comparative oncologic safety and treatment-related outcomes between these two methods of reconstruction.

## 5. Conclusions

The existing evidence shows that PAP flap reconstruction represents a reliable alternative to DIEP flap breast reconstruction (and is increasingly integrated into clinical practice), achieving comparable flap survival rates, acceptable donor-site morbidity, and favorable patient-reported outcomes in appropriately selected cases. Although DIEP reconstruction remains the gold standard for autologous breast reconstruction, PAP flaps provide a valuable reconstructive option, particularly in patients with low BMI or insufficient abdominal tissue availability.

Currently, the available literature on PAP reconstruction is mainly focused on surgical outcomes, complication profiles, and aesthetic or patient-reported measures. In contrast, DIEP reconstruction has been widely researched with respect to reconstructive timing, oncologic safety, recurrence, and the consequences of radiotherapy and chemotherapy. Existing evidence indicates that immediate DIEP reconstruction is oncologically safe, with low recurrence rates, and in line with modern multimodal oncologic treatment strategies.

Still, a major gap persists concerning PAP reconstruction in oncologic settings. No currently available study directly assessed immediate versus delayed PAP reconstruction, nor compared PAP and DIEP flaps with respect to reconstructive timing, radiotherapy exposure, chemotherapy, or long-term oncologic outcomes. Therefore, current clinical decision-making encompassing PAP flaps continues to rely extensively on extrapolation from DIEP-based evidence.

Whether PAP flap reconstruction matches the oncologic safety profile of DIEP reconstruction across immediate and delayed timing and during adjuvant oncologic treatment remains an open question.

## Figures and Tables

**Table 1:** Overview and classification of studies included in the narrative review.

Category	Included Studies	Number of Studies
PAP vs. DIEP/alternative donor sites	Charles et al., 2024 [1]; Chowdhury et al., 2024 [2]; Zhu & Liu, 2024 [3]; Augustin et al., 2023 [4]; Valenzuela, 2025 [5]; Haddock et al., 2024 [6]; Minkhorst et al., 2024 [7]; Varnava et al., 2023 [8]; Atzeni et al., 2021 [9]; Kim et al., 2022 [10]; Lu et al., 2023 [20].	11
DIEP timing (immediate vs. delayed)	Prantl et al., 2020 [11]; Wu et al., 2023 [12]; Nguyen et al., 2023 [13]; Nava et al., 2025 [14]; Jansen et al., 2025 [15]; Joosen et al., 2021 [16]; Lee et al., 2023 [17]; Alves et al., 2022 [18].	8
Oncologic/RT-CT related outcomes	Prantl et al., 2020 [11]; Jansen et al., 2025 [15]; Joosen et al., 2021 [16]; Nguyen et al., 2023 [13]; Nava et al., 2025 [14]; Alves et al., 2022 [18]; Abdallah et al., 2024 [19].	7

**Table 2:** Main Direct Comparative Studies Between PAP and DIEP Flap Breast Reconstruction.

Study	Study Design/Population	Comparison	Main Findings	Key Conclusion
Charles et al., 2024 [1]	Retrospective comparative study; 677 patients	559 DIEP vs. 118 PAP	PAP patients were younger and had smaller breast volumes. No significant differences in operative time, reoperations, or overall complications.	PAP flap might represent a primary reconstructive option rather than only a secondary alternative.
Chowdhury et al., 2024 [2]	Prospective cohort; 157 patients	DIEP vs. PAP	PAP was linked to shorter ischemia time but higher donor-site wound dehiscence. One-year BREAST-Q outcomes were comparable between groups.	Patient-reported outcomes were similar despite differing donor-site complication profiles.
Augustin et al., 2023 [4]	Matched prospective study; 36 patients	DIEP vs. PAP	No significant differences in BREAST-Q scores, scar quality, donor-site morbidity, or breast aesthetics.	Long-term patient-reported and aesthetic outcomes were comparable between techniques.
Haddock et al., 2024 [6]	Propensity-matched retrospective comparison; 150 patients	50 DIEP vs. 50 PAP vs. 50 LAP	Long-term satisfaction was similar across groups. PAP had more donor-site wound complications, while DIEP had more breast wound complications and flap necrosis.	Different flap types demonstrated distinct complication patterns despite comparable satisfaction outcomes.
Varnava et al., 2023 [8]	Retrospective single-center study; 207 patients	85 PAP vs. 122 DIEP	PAP reconstruction showed shorter operative time and faster ambulation, while DIEP achieved higher BREAST-Q scores.	PAP reconstruction has substantial potential but may require further refinement compared with DIEP.
Kim et al., 2022 [10]	Retrospective ultrasound-based study; 130 reconstructions	31 PAP vs. 99 DIEP	No total flap loss occurred in either group. PAP reconstruction demonstrated higher donor-site morbidity and higher rates of fat necrosis.	PAP flap reconstruction may be associated with increased donor-site complications and fat necrosis.

DIEP = deep inferior epigastric perforator flap; PAP = profunda artery perforator flap; BREAST-Q = breast reconstruction questionnaire; LAP = lumbar artery perforator flap.

**Table 3:** PAP flap outcomes from meta-analyses, systematic reviews, and clinical series.

Study	Design/Population	Key Findings	Comparison/Context
Zhu & Liu, 2024 [3]	Meta-analysis (24 studies; 1612 PAP reconstructions)	Success rate 99.6%; total flap loss 0.4%; infection 0.3%; hematoma 1.4%; seroma 1.0%; fat necrosis 3.3%; donor-site dehiscence 9.2%	No significant differences compared to DIEP in flap failure, fat necrosis, or donor-site dehiscence (where reported); PAP patients were younger and had lower BMIs
Minkhorst et al., 2024 [7]	Systematic review (PAP/LAP vs. DIEP)	PAP success 98.3% vs. DIEP 98.4%; low fat necrosis; donor-site dehiscence 9.1%; stacked PAP and LAP had lower survival than single flaps	DIEP vs. PAP vs. LAP comparison; overall similar survival for single PAP and DIEP
Atzeni et al., 2021 [9]	Prospective single-center series (116 PAP reconstructions)	No flap loss; hematoma 1.7%, seroma 2.6%, fat necrosis 1.7%, dehiscence 2.6%; significant BREAST-Q improvement at 12 months	PAP-only outcomes with favorable functional and QoL results
Lu et al., 2023 [20]	Narrative clinical review	Their findings consolidate PAP flap anatomy, surgical technique, and clinical outcomes, demonstrating a low complication profile across published series	The authors position the PAP flap as a strong alternative when DIEP flap reconstruction is not feasible

PAP = profunda artery perforator flap; DIEP = deep inferior epigastric perforator flap; LAP = lumbar artery perforator flap; BREAST-Q = breast reconstruction questionnaire; BMI = body mass index.

**Table 4:** DIEP flap timing, oncologic safety, and treatment-related outcomes.

Study	Design/Population	Focus	Key Findings
Prantl et al., 2020 [11]	Multicenter cohort (3926 patients; 4577 DIEPs)	Immediate vs. delayed DIEP	Similar flap loss rates in IBR vs. DBR; IBR had fewer revisions, shorter hospitalization, and earlier mobilization
Wu et al., 2023 [12]	Retrospective cohort (802 DIEPs)	Risk factors	Overall complications 15.5%; longer OR time, with increased complication risk for immediate reconstruction
Nguyen et al., 2023 [13]	Prospective cohort (30 DIEPs)	Immediate DIEP outcomes	Flap success rate 90%; complications included flap loss (6.7%) and venous congestion (13.3%); CT start ~35 days
Nava et al., 2025 [14]	Retrospective cohort (141 DIEPs)	RT/CT impact	Preop RT + CT increased complications; ischemia time independently increased the rate of recipient-site complications
Jansen et al., 2025 [15]	Multicenter PRO cohort (88 patients)	DIEP + PMRT	Early QoL is better in IBR; long-term PROs similar; more fibrosis and mobility restriction after IBR
Joosen et al., 2021 [16]	Cohort (862 patients; 919 DIEPs)	Recurrence in the case of IBR vs. DBR	Local recurrence rate ~1.5–1.7%; higher recurrence risk in DBR is suggested but likely due to selection bias
Lee et al., 2023 [17]	Large cohort (1474 reconstructions)	TE/I vs. DIEP + RT	DIEP had lower 5-year complications vs. TE/I, especially in patients with RT; no difference in the CT-only subgroup
Alves et al., 2022 [18]	Meta-analysis (4 studies; 5784 DIEPs)	IBR vs. DBR complications	IBR group—reduced wound-healing issues (OR: 0.57); no differences regarding infection, hematoma, fat necrosis, flap loss
Abdallah et al., 2024 [19]	Cohort (666 DIEPs)	Local recurrence	Overall recurrence was 2%, with no evidence of increased local recurrence risk compared with mastectomy alone; lap preservation was frequently feasible.

TE/I = tissue expander/implant reconstruction; IBR = immediate breast reconstruction; DBR = delayed breast reconstruction; RT = radiotherapy; CT = chemotherapy; PMRT = postmastectomy radiotherapy; OR = operating room; PROs = patient-reported outcomes; QoL = quality of life; TE = tissue expander; DIEP = deep inferior epigastric perforator flap.

## Data Availability

No new data were created or analyzed in this study.
